# Informed citizen and empowered citizen in health: results from an European survey

**DOI:** 10.1186/1471-2296-12-20

**Published:** 2011-04-16

**Authors:** Silvina Santana, Berthold Lausen, Maria Bujnowska-Fedak, Catherine E Chronaki, Hans-Ulrich Prokosch, Rolf Wynn

**Affiliations:** 1 Institute of Electronics Engineering and Telematics of Aveiro and Department of Economics, Management and Industrial Engineering, University of Aveiro, Aveiro, Portugal; 2 Department of Mathematical Sciences, University of Essex, Colchester, UK; 3 Department of Family Medicine, Wrocław Medical University, Wrocław, Poland; 4 Institute of Computer Science, Foundation for Research & Technology-Hellas (FORTH), Heraklion, Crete, Greece; 5 Chair of Medical Informatics, Friedrich-Alexander-University Erlangen-Nürnberg, Erlangen, Germany; 6 Institute of Clinical Medicine (FT), University of Tromsø, Norway and Division of Substance Abuse and Specialized Psychiatry, University Hospital of North Norway, Tromsø, Norway

## Abstract

**Background:**

The knowledge about the relationship between health-related activities on the Internet (i.e. informed citizens) and individuals' control over their own experiences of health or illness (i.e. empowered citizens) is valuable but scarce. In this paper, we investigate the correlation between four ways of using the Internet for information on health or illness and citizens attitudes and behaviours toward health professionals and health systems and establish the profile of empowered eHealth citizens in Europe.

**Methods:**

Data was collected during April and May 2007 (N = 7022), through computer-assisted telephone interviews (CATI). Respondents from Denmark, Germany, Greece, Latvia, Norway, Poland and Portugal participated in the survey. The profiles were generated using logistic regressions and are based on: a) socio-demographic and health information, b) the level of use of health-related online services, c) the level of use of the Internet to get health information to decide whether to consult a health professional, prepare for a medical appointment and assess its outcome, and d) the impact of online health information on citizens' attitudes and behavior towards health professionals and health systems.

**Results:**

Citizens using the Internet to decide whether to consult a health professional or to get a second opinion are likely to be frequent visitors of health sites, active participants of online health forums and recurrent buyers of medicines and other health related products online, while only infrequent epatients, visiting doctors they have never met face-to-face. Participation in online health communities seems to be related with more inquisitive and autonomous patients.

**Conclusions:**

The profiles of empowered eHealth citizens in Europe are situational and country dependent. The number of Europeans using the Internet to get health information to help them deal with a consultation is raising and having access to online health information seems to be associated with growing number of inquisitive and self-reliant patients. Doctors are increasingly likely to experience consultations with knowledgeable and empowered patients, who will challenge them in various ways.

## Background

Empowerment [1-4] is a frequently used and sometimes abused word entailing an ideal increasing in popularity and application in many domains and particularly in health [5,6]. Yet the knowledge available on the dynamics of empowerment of societies [7] or specific groups [8] in the health domain is scarce and the link between online access to health information and empowerment needs further empirical investigation [9].

Empowerment has been defined as the enhancement of "the possibility for people to control their own lives" [4,15]. Empowerment and particularly patient empowerment engages individuals, groups, organizations, communities and governments while it requires the ability to gain control over many aspects of individuals' lives. For citizens it implies individual responsibility in health care, whereas for communities it implies the broader health professional, group, organizational, institutional and societal role in enabling citizens to assume responsibility for their own health, as individuals and as communities.

Empowerment might be seen as both a process and as the outcome of a process [10]. Empowering processes for individuals might include "participation in community organizations", while empowered outcomes for individuals might include "situation-specific perceived control and resource mobilization skills" [[11]:570-571]. Recent conceptual model of empowerment for application to a general patient population [12] represents empowerment as a continuous process based on antecedents, or the elements that allow patients to start the empowerment process (knowledge, health literacy, patient initiative, access to services); processes (information sharing, patient-doctor communication, choice, shared decision making, patient self-care); and outcomes for the patient (health related outcomes, satisfaction, self-efficacy, adherence, control over her/his health, care-seeking behaviour, understanding when medical attention is necessary) [13]. However, there is no generally accepted definition of empowerment nor agreement on how to conduct its measurement [14], especially when the Internet and the Web are involved [15]. Often, assessment instruments are disease specific [16,17] and many measure only one or some empowerment related outcomes [18,19]. Globally, few instruments measure a patient's degree of individual empowerment in relation to personal health care and services [20].

Empowerment can promote the goals of patients but also the goals of other stakeholders [21]. The emergence of the "empowered patient" concept coincides in time with a steep increase in health care costs in most Western countries and governments' attempt to reduce health care expenditures with the correspondent transfer of responsibility for health care to individuals [22]. These trends emerged in a context of increasing access to the Internet and its use for health and illness matters. Such pressures, resources and opportunities may be fuelling other significant social trends, as the expansion of self and mutual aid programs focused on the patient, the growth of a certain consumerism associated to the topic of health, the increase of patients' organization and activism and the turn towards complementary and alternative medicine [22]. All these may have significant impact on the way citizens assume responsibility for their own health and, consequently, on their relationship with health professionals and the health system.

The essence of empowerment is personal control, inextricably linked to available information, acquired knowledge and capacity to learn. Traditionally, the doctor-patient relationship evolves in a context of considerable power imbalance, where the doctor possesses medical knowledge, and patient knowledge is often considered irrelevant [23,24]. This competence gap contributes to maintaining patient dependency [25].

One of the underlying assumptions of patient empowerment is precisely that providing health information to patients empowers them [22]. However, such a straightforward approach detracts from a complex reality involving not only the patient, the physician, the particular conditions that brought them together and the relationship they develop at each appointment and over time but also legal, cultural, and educational aspects that are specific to different societies and evolve over time [26].

Most citizens will experience the need for healthcare at some time and many interact with the health system on behalf of somebody else [27]. They participate in society making decisions about health or passively accepting the decisions of others. Objective health information comes from several sources including doctors, patient organizations, National Health Agencies, pharmaceutics [28], and lately the Internet.

Even accepting that "the principal route to an informed patient is the patient-doctor meeting (the clinical consultation)" [28,1] and knowing that the physician is still considered by citizens as the most important [29] source of generic health information and the most accurate [30] source of information on mental health issues, the increasing relevance of the Internet is undeniable, and so is its potential to change the patient-doctor relationship [31]. Medical professionals and researchers no longer control the production and dissemination of health information and citizens now have access to electronic versions of medical journals and other online sources of health related information [23]. Citizens have become co-producers of health information that is spread through email and virtual communities, a phenomenon often cited as "empowering" [22,32] and reading in itself seems sufficient to profit from participation in online patient support groups [33]. Communicating online seeking advice from doctors they have never met [34], getting suggestions or recommendations from other patients, and ordering medicine are other opportunities to become informed. Many state that the medical information and guidance they can find online is more complete and useful than the information that is typically provided by their physicians [35]. General practitioners report that the length of consultation is increased due to patient questions relevant to information found on the Internet and that patients holding Internet healthcare information have higher expectations [36].

Evidence on actual use of the Internet for health purposes is mixed. In Europe, it varies from one country to another, but the perceived importance of the technology is rising and recent work suggests that interactive use is increasing [21,29,37]. In the US in 2009, 61% of American adults had looked online for health information and around one third had accessed social media related to health [38]. In 2005, 10% of Internet users reported communicating online with an healthcare provider [39] and in 2004, 4% have bought prescription drugs on the Internet [40]. In a national survey conducted in 2003, 55% of Internet health information seekers reported having consulted a health professional because of the information they received online [41]. A study comparing data from 2000 and 2002 shows that the gap for the access to online health information between old and young people tends to increase, even as literature continue to promote the Internet as a key source of empowerment for the elderly [42]. Therefore, investigating if and how the Internet is giving citizens more control over their experiences of health or illness and whether it is changing their relationships with health professionals and health systems becomes crucial.

Better informed and knowledgeable patients as a result of accessing information from health sites and health communities may be better prepared and likely to ask doctors relevant and critical questions [43,44]. Information on drugs and treatments may lead to pressure on health professionals to provide access to other options [5,8,23,45]. Governments and medical professionals fear that patients may use the Internet to avoid doctors altogether, which perceivably could lead to worsened health [23] while others [[46]:174] consider the use of some models of online pharmacies "a perfect illustration of the inherent shortcomings of consumer empowerment initiatives that rely on markets without implementing appropriate regulation mechanisms".

In this paper, we explore data originating from the second WHO/European eHealth Consumer Trends Survey (eHealth Trends survey). Previous work [37] based on the first eHealth Trends survey conducted in 2005 has shown that, in the general population, 29% have turned to the Internet to find health information to decide whether to consult a health professional, 23% to find health information prior to an appointment and 27% to find health information after an appointment; 20% have made suggestions or queries on diagnosis or treatment to a health professional, 2% have changed the use of medicine without consulting a health professional and 6% have made/cancelled/changed a consultation as a result of health information from the Internet.

In the present work, we go deeper in the analysis, investigating the correlation between four Internet activities that might foster the process of empowerment by increasing health knowledge, health literacy, patient initiative and access to services - important antecedents of the process [13], and two outcomes of this process directly connected to attitudes toward the practice of health professionals and health system.

First, empowerment that enables the individual to be active in looking for information that gives him/her more autonomy and reinforces his/her position when relating with health professionals, namely deciding whether to get an appointment, preparing for the consultation and validating its outcome [[10]:583]. Second, empowerment that translates into behaviour that directly challenges the authority and autonomy of health professionals, namely making suggestions or queries on diagnosis or treatment, changing the use of medicine without consulting a health professional and (re)scheduling an appointment with a health professional.

The four online activities we explore are: interacting with health professionals never met face to face, participating in forums or self-help groups focusing on health or illness, ordering medicine or other products related to health or illness management and reading about health and illness. Efforts to gain control, access to resources, and a critical understanding of one's socio-political context are fundamental aspects of empowering processes [10]. Therefore, in the context of this work we expect those performing such Internet activities to be more active in looking for information to help them deal with a consultation and to exhibit behaviour more challenging to the traditional view of authority and autonomy of health professionals [23].

## Methods

### Study design

We postulated that citizens are using online services to support their decision on whether they need a medical appointment, to prepare for it, and to analyze its outcome. We hypothesized that having access to health information through online services gives rise to concrete types of behaviour towards health professionals and health systems, namely making suggestions or queries on diagnosis or treatment, changing the use of medicine without consulting a health professional and making, cancelling or changing a medical appointment.

Citizens from Denmark, Germany, Greece, Latvia, Norway, Poland and Portugal were selected according to a stratified sampling plan developed for each country and interviewed during April and May 2007, aiming at a representative sample of 7000 usable interviews (for more details about the all project see [37] and [29]). National ethics committees were informed and had no objections to the survey.

### Procedures

The frequency of online activities intended to get information related to health or illness was assessed by Question A: "How often do you use the Internet to: 1) interact with health professionals you have never met face to face; 2) participate in forums or self-help groups (focusing on health or illness); 3) order medicine or other products related to health or illness management online; 4) read about health and illness". The response categories were "Every day", "Every week", "Every month", "Every six months", "Every year", "Less than once a year" and "Never". The responses were recoded into "Never", "Infrequent" ("Every six months", "Every year", "Less than once a year") and "Established" ("Every day", "Every week", "Every month") in order to be used as independent variables in logistic regression models.

As outcomes of the process of empowerment we explore two situations: being active in looking for online health information in order to be in a better position when dealing with a consultation, namely when deciding whether to get an appointment, preparing for the consultation and validating its outcome; and reporting behaviour that directly challenges the authority and autonomy of health professionals, namely making suggestions or queries on diagnosis or treatment, changing the use of medicine without consulting a health professional and (re)scheduling an appointment with a health professional.

How the Internet was used to get health information to better handle a consultation, was measured by Question B: "Do you use the Internet to 1) find health information that can help you decide whether to consult a health professional; 2) find health information prior to an appointment; 3) find information after an appointment with health professionals (e.g. for second opinion)". The response categories were "Always", "Often", "Sometimes", "Rarely" and "Never". We believe that the most important distinction is whether the patient has actually used the Internet for these purposes or not and the responses were therefore recoded into 0 (never used) and 1 (have used), in order to be used in logistic regressions. Detailed data analysis has confirmed the legitimacy of this assumption.

Behaviours towards health professionals and health systems fostered by online information were assessed by Question C: "Has information on health or illness which you have obtained from the Internet led to any of the following: 1) Suggestions or queries on diagnosis or treatment to your family doctor, specialist or other health professional; 2) Changing the use of medicine without consulting your family doctor, specialist or other health professional; 3) Making, cancelling or changing an appointment with your family doctor, specialist or other health professional". The response categories were "Yes", "No" and "Do not know". When using the correspondent variables as dummy dependent variables in logistic regressions, "Do not know" answers were recoded as missing values and excluded from the complete case analysis.

The questionnaire was designed in English with the collaboration of researchers from the seven participating countries. To ensure internal validity and comprehensibility of wording, the instrument was piloted on 100 individuals in Norway. Afterwards, it was translated to the language or languages of the participating countries using a dual focus method [47] that aims at conceptual equivalence, beside dealing with grammar and wording aspects. Within the translation procedure, focus groups were used to refine and evaluate the final instrument. Data was collected by poll agencies through Computer-Assisted Telephone Interview (CATI).

The sampling plan for each country was developed considering gender and age (six groups). Random digit dialling in strata ensured a randomized representative sample of the seven countries populations. With this procedure, sampling continues until a previously defined number of complete interviews is achieved. Therefore, a country-dependent number of calls were made until having approximately 1000 complete interviews from all countries. In total, 7022 questionnaires were completed, corresponding to an average response rate of 36% of the 22867 individuals contacted in the seven countries (for more details see [29]). Overall, no variables had more than 5% missing data.

### Statistical analysis

Tables 1 and 2 provide observed frequencies and mean percentages for the year 2007 and change, in mean percentage, between the year 2007 and the year 2005 (weighted data). Tables 1 and 2 also provide 95% confidence intervals derived from Gaussian approximations of the distribution of the sum of strata frequencies or sum of ratios of strata frequencies. P-values of two-sided tests are not given. For one specific test results are reported in italic when the null is not inside the 95% interval. Differences (2007 minus 2005) were computed using post-stratified data of the first eHealth Trends survey (October-November 2005) in the analyses (Tables 1 and 2). Post-stratified weighting of the 2005 distribution was defined by weights based on the 2007 distribution regarding six age groups (15-25, 26-35, 36-45, 46-55, 56-65 and 66-80 years) by gender, in order to separate real effects from minor changes introduced by sample construction (for more details see [29]).

**Table 1:** Observed frequency and percentage of citizens turning to the Internet to find health information that might help them deal with a consultation in 2007 and changes from 2005 to 2007

	**2007**			**Change from 2005 to 2007**		
		
	**Find health information to help decide whether to consult a health professional**	**Find health information prior to an appointment**	**Find information after an appointment with health professionals**	**Find health information to help decide whether to consult a health professional**	**Find health information prior to an appointment**	**Find information after an appointment with health professionals**
		
	**Frequency**	**Frequency**	**Frequency**			
	**Mean % (CI)**^**a**^	**Mean % (CI)**^**a**^	**Mean % (CI)**^**a**^	**Mean % (CI)**^**a**^	**Mean % (CI)**^**a**^	**Mean % (CI)**^**a**^
	471	348	364			
Denmark	46.1(43.2-49.0)	34.1(31.3-36.9)	35.7(32.8-38.5)	*13.8(9.7-17.8)*	*7.5(3.6.-11.4)*	*5.3(1.3-9.3)*
	413	261	357			
Germany	41.3 (38.5-44.1)	26.1(23.5-28.7)	35.7(32.9-38.5)	*9.5(17.2-3.7)*	*3.7(0.0-7.3)*	*8.2(4.3-12.2)*
	236	210	208			
Greece	23.6(21.2-26.0)	21.0(18.6-23.4)	20.8(18.4-23.2)	*13.0(9.6-6.5)*	*9.6(6.5-12.7)*	*9.1(6.1-12.2)*
	303	228	253			
Latvia	30.3(27.7-32.9)	22.8(20.4-25.2)	25.3(22.8-27.8)	*9.1(5.7-12.5)*	*6.8(3.6-10.1)*	*10.2(7.0-13.5)*
	390	278	358			
Norway	39.0(36.2-41.7)	27.8(25.2-30.4)	35.8(33.0-38.6)	*5.3(1.4-9.3)*	3.2(-0.5-7.0)	*4.9(0.9-8.9)*
	383	315	335			
Poland	38.3(35.6-41.0)	31.5(28.9-34.1)	33.5(30.8-36.2)	*7.0(3.3-10.8)*	*5.1(1.5-8.7)*	*5.9(2.2-9.6)*
	189	156	177			
Portugal	18.9(16.6-21.2)	15.6(13.5-17.7)	17.7(15.4-20.0)	*5.8(3.0-8.5)*	*3.5(0.9-6.1)*	*5.3(2.6-8.0)*
	2385	1796	2052			
Total	33.9(32.9-34.9)	25.6(24.6-26.5)	29.2(28.2-30.2)	*9.2(7.8-10.5)*	*5.6(4.3-6.9)*	*7.0(5.7-8.4)*

^a^95% confidence intervals (CI); differences are typed in italic when significantly different from 0 at the 5% level

**Table 2:** Observed frequency and percentage of citizens that have made suggestions or queries on diagnosis or treatment to a health professional or have taken health decisions as a result of health information from the Internet in 2007 and changes from 2005 to 2007

	**2007**			**Change from 2005 to 2007**		
		
	**Suggestions or queries on diagnosis or treatment to health professional**	**Changing the use of medicine without consulting a health professional**	**Making, cancelling or changing an appointment with health professional**	**Suggestions or queries on diagnosis or treatment to health professional**	**Changing the use of medicine without consulting a health professional**	**Making, cancelling or changing an appointment with health professional**
		
	**Frequency**	**Frequency**	**Frequency**			
	**Mean % (CI)**^**a**^	**Mean % (CI)**^**a**^	**Mean % (CI)**^**a**^	**Mean % (CI)**^**a**^	**Mean % (CI)**^**a**^	**Mean % (CI)**^**a**^
	
	251	30	82			
Denmark	24.6(22.0-27.2)	2.9(1.9-4.0)	8.0(6.4-9.7)	*4.7(1.1-8.3)*	-0.1(-1.7-1.4)	2.0(-0.2-4.3)
	268	34	64			
Germany	26.8(24.2-29.4)	3.4(2.3-4.5)	6.4(4.9-7.9)	*7.5(4.0-11.1)*	1.1(-0.5-2.5)	1.3(-0.7-3.3)
	152	9	30			
Greece	15.2(13.0-17.4)	0.9(0.3-1.5)	3.0(2.0-4.0)	2.2(-0.8-5.1)	0.4(-0.4-1.1)	*1.3(0.0-2.6)*
	276	62	99			
Latvia	27.6(25.0-30.2)	6.2(4.7-7.7)	9.9(8.1-11.7)	*7.6(4.2-11.1)*	1.8(-0.2-3.7)	*2.4(0.0-4.9)*
	166	30	83			
Norway	16.6(14.3-18.8)	3.0(1.9-4.0)	8.3(6.6-10.0)	*3.7(0.6-6.8)*	0.4(-1.1-1.8)	1.4(-1.0-3.8)
	281	35	111			
Poland	28.1(25.5-30.7)	3.5(2.4-4.6)	11.1(9.2-13.0)	*6.4(2.9-9.9)*	1.1(-0.5-2.5)	*4.0(1.5-6.4)*
	151	20	28			
Portugal	15.1(13.0-17.2)	2.0(1.1-2.9)	2.8(1.8-3.8)	*2.8(0.2-5.4)*	0.9(-0.1-1.9)	0.1(-1.2-1.3)
	1545	220	497			
Total	22.0(21.1-22.9)	3.1(2.7-3.5)	7.1(6.5-7.7)	*5.0(3.7-6.2)*	*0.7(0.2-1.3)*	*1.8(1.0-2.6)*

^a^95% confidence intervals (CI); differences are typed in italic when significantly different from 0 at the 5% level

Factors associated with looking for health information to help deal with a consultation and factors with impact on citizens' behaviour toward health professionals and health systems were tested by a series of logistic regressions, in which the dichotomous outcomes of Questions B and C were regressed on demographic, socioeconomic and health variables, as well as the outcomes of Question A. For each factor level the odds ratio and 95% confidence intervals of the odds ratios were reported. Factors are tested with type II hypotheses (function Anova, R package: car version 1.2-7). Overall, nine multivariate models were tested (Tables 3, 4, 5, 6, 7). All analyses were performed with SPSS (version 16.0) and R [48] (version 2.8.1).

**Table 3:** Factors associated with looking for health information to help deal with a consultation among general population

	Find health information to help decide whether to consult a health professional			Find health information prior to an appointment			Find information after an appointment with health professionals		
	Oddsratio	CI (95%)	*P*-value	Oddsratio	CI (95%)	*P*-value	Oddsratio	CI (95%)	*P*-value
**Male**	1.02	0.88-1.19	0.767	0.89	0.77-1.02	0.103	0.93	0.8-1.08	0.328
Female	1			1			1		
**Age**			*<.001*			*<.001*			*0.034*
15-25	4.41	2.86-6.79	*<.001*	2.48	1.61-3.82	*<.001*	1.64	1.08-2.48	*0.02*
26-35	3.77	2.52-5.65	*<.001*	2.44	1.62-3.68	*<.001*	1.75	1.18-2.58	*0.005*
36-45	2.74	1.82-4.13	*<.001*	2.1	1.38-3.19	*<.001*	1.73	1.16-2.58	*0.007*
46-55	2.25	1.5-3.38	*<.001*	1.59	1.05-2.41	*0.029*	1.46	0.98-2.17	0.06
56-65	1.68	1.13-2.51	*0.011*	1.11	0.73-1.69	0.632	1.21	0.82-1.8	0.332
66-80	1			1			1		
**Education**			*0.021*			*<.001*			*<.001*
Higher education	1.38	1.09-1.75	*0.007*	2.07	1.64-2.61	*<.001*	2.04	1.62-2.56	*<.001*
A-Level	1.29	1.04-1.59	*0.019*	1.38	1.12-1.7	*0.003*	1.33	1.08-1.63	*0.007*
Below A-Level	1			1			1		
**Kids at home (< 18)**	1.04	0.88-1.23	0.638	0.99	0.84-1.16	0.897	1	0.85-1.17	0.995
No kids at home	1			1			1		
**Place of living**			0.086			*0.002*			*0.020*
City	1.32	1.02-1.72	*0.034*	1.64	1.26-2.14	*<.001*	1.5	1.16-1.94	*0.002*
Minor city or suburbs of a big city	1.41	1.08-1.85	*0.011*	1.51	1.15-1.97	*0.003*	1.35	1.04-1.76	*0.022*
Village	1.32	1.01-1.73	*0.045*	1.59	1.21-2.09	*<.001*	1.35	1.03-1.76	*0.028*
Rural/scattered house	1			1			1		
**Work situation**			0.43			0.361			0.643
Student	1.24	0.89-1.71	0.199	1.17	0.87-1.59	0.302	1.16	0.85-1.56	0.35
Working	1.09	0.88-1.35	0.425	0.96	0.78-1.19	0.735	1.04	0.84-1.28	0.711
Not at work	1			1			1		
**Disable or with diagnosis**	1.05	0.83-1.34	0.665	0.98	0.78-1.24	0.871	1.18	0.94-1.49	0.16
No	1			1			1		
**Relative disable or with diagnosis**	1.03	0.87-1.22	0.736	0.94	0.8-1.11	0.473	1	0.85-1.18	0.977
No	1			1			1		
**Subjective health**			0.718			0.661			0.829
Good	0.84	0.54-1.29	0.426	0.83	0.54-1.25	0.368	0.95	0.63-1.44	0.815
Fair	0.86	0.56-1.34	0.514	0.83	0.54-1.27	0.383	1.01	0.66-1.53	0.976
Bad	1			1			1		
**One or more visits to GP in the previous year**	1.13	0.92-1.39	0.244	1.3	1.06-1.6	*0.012*	1.55	1.26-1.89	*<.001*
No visits to GP in the previous year	1			1			1		
**Use of the Internet to interact with unknown HP**			*<.001*			*<.001*			*<.001*
Established	1.34	0.92-1.94	0.123	1.19	0.87-1.63	0.284	1.4	1-1.95	0.052
Infrequent	2.11	1.7-2.62	*<.001*	1.72	1.43-2.08	*<.001*	1.78	1.47-2.16	*<.001*
No	1			1			1		
**Use of the Internet to participate in forums or self help groups**			*<.001*			*<.001*			*<.001*
Established	1.87	1.26-2.77	*0.002*	1.65	1.21-2.24	*0.002*	1.72	1.23-2.39	*0.001*
Infrequent	1.69	1.33-2.13	*<.001*	1.92	1.56-2.36	*<.001*	1.66	1.34-2.05	*<.001*
No	1			1			1		
**Use of the Internet to order medicines or other products related to health**			*<.001*			*<.001*			*<.001*
Established	2.13	1.29-3.51	*0.003*	1.88	1.27-2.79	*0.002*	2	1.3-3.08	*0.002*
Infrequent	1.54	1.22-1.94	*<.001*	1.34	1.09-1.65	*0.006*	1.33	1.07-1.64	*0.009*
No	1			1			1		
**Use of the Internet to read about health and illness**			*<.001*			*<.001*			*<.001*
Established	63.02	48.49-81.92	*<.001*	67.09	47.49-94.77	*<.001*	65.19	48.43-87.75	*<.001*
Infrequent	31.73	24.62-40.88	*<.001*	35.54	25.19-50.14	*<.001*	35.74	26.65-47.94	*<.001*
No	1			1			1		

*P-values *are typed in italic when significant at the 5% level

**Table 4:** Factors associated with looking for health information to help deal with a consultation in the subgroup of Internet users for health or illness matters

	Find health information to help decide whether to consult a health professional			Find health information prior to an appointment			Find information after an appointment with health professionals		
	Oddsratio	CI (95%)	*P*-value	Oddsratio	CI (95%)	*P*-value	Oddsratio	CI (95%)	*P*-value
**Male**	0.99	0.85-1.15	0.863	0.87	0.75-1.0	0.058	0.9	0.78-1.04	0.146
Female	1			1			1		
**Age**			*<.001*			*<.001*			0.173
15-25	3.67	2.33-5.78	*<.001*	2.14	1.37-3.34	*<.001*	1.31	0.84-2.02	0.234
26-35	3.29	2.15-5.03	*<.001*	2.16	1.41-3.29	*<.001*	1.43	0.94-2.17	0.095
36-45	2.41	1.57-3.72	*<.001*	1.86	1.2-2.87	*0.005*	1.42	0.93-2.18	0.106
46-55	2	1.3-3.07	*0.001*	1.42	0.92-2.18	0.112	1.21	0.79-1.85	0.379
56-65	1.49	0.97-2.28	0.066	0.99	0.64-1.53	0.966	1.01	0.66-1.55	0.949
66-80	1			1			1		
**Education**			0.354			*<.001*			*<.001*
Higher education	1.19	0.93-1.52	0.157	1.92	1.52-2.44	*<.001*	1.82	1.44-2.3	*<.001*
A-Level	1.15	0.92-1.43	0.219	1.31	1.06-1.62	*0.012*	1.23	0.99-1.51	0.056
Below A-Level	1			1			1		
**Kids at home (< 18)**	1.02	0.86-1.2	0.847	0.98	0.84-1.15	0.81	0.98	0.84-1.15	0.85
No kids at home	1			1					
**Place of living**			0.077			*0.001*			*0.010*
City	1.36	1.05-1.77	*0.02*	1.67	1.28-2.17	*<.001*	1.54	1.2-1.99	*<.001*
Minor city or suburbs of a big city	1.42	1.08-1.85	*0.011*	1.51	1.16-1.97	*0.002*	1.36	1.05-1.77	*0.019*
Village	1.33	1.01-1.74	*0.042*	1.59	1.21-2.09	*<.001*	1.36	1.04-1.77	*0.025*
Rural/scattered house	1			1			1		
**Work situation**			0.896			0.438			0.876
Student	1.08	0.78-1.5	0.643	1.11	0.82-1.5	0.508	1.07	0.79-1.45	0.66
Working	1.02	0.82-1.27	0.865	0.94	0.75-1.16	0.54	1	0.81-1.24	0.994
Not at work	1			1			1		
**Disable or with diagnosis**	1.03	0.81-1.31	0.819	0.97	0.77-1.22	0.777	1.16	0.92-1.47	0.215
No	1			1			1		
**Relative disable or with diagnosis**	1.05	0.88-1.25	0.605	0.95	0.81-1.12	0.534	1.01	0.86-1.19	0.874
No	1			1			1		
**Subjective health**			0.535			0.614			0.592
Good	0.81	0.51-1.28	0.359	0.81	0.53-1.24	0.325	0.92	0.6-1.41	
Fair	0.87	0.55-1.39	0.559	0.83	0.54-1.27	0.387	1.01	0.65-1.56	0.965
Bad	1			1			1		
**One or more visits to GP in the previous year**	1.16	0.94-1.42	0.156	1.32	1.08-1.62	*0.008*	1.57	1.28-1.92	*<.001*
No visits to GP in the previous year	1			1					
**Use of the Internet to interact with unknown HP**			*<.001*			*<.001*	1		*<.001*
Established	1.22	0.85-1.75	0.277	1.14	0.83-1.55	0.414	1.31	0.94-1.82	0.11
Infrequent	1.84	1.5-2.26	*<.001*	1.63	1.36-1.96	*<.001*	1.64	1.36-1.99	*<.001*
No	1			1			1		
**Use of the Internet to participate in forums or self help groups**			*<.001*			*<.001*			*<.001*
Established	1.82	1.24-2.67	*0.002*	1.64	1.21-2.23	*0.002*	1.7	1.23-2.35	*0.001*
Infrequent	1.61	1.28-2.02	*<.001*	1.87	1.53-2.29	*<.001*	1.61	1.31-1.98	*<.001*
No	1			1			1		
**Use of the Internet to order medicines or other products related to health**			*<.001*			*<.001*			*<.001*
Established	1.98	1.23-3.19	*0.005*	1.84	1.25-2.71	*0.002*	1.92	1.26-2.91	*0.002*
Infrequent	1.45	1.16-1.81	*0.001*	1.31	1.07-1.61	*0.009*	1.28	1.04-1.58	*0.018*
No	1			1			1		
**Use of the Internet to read about health and illness**			*<.001*			*<.001*			*<.001*
Established	5.53	4.11-7.42	*<.001*	6.78	4.72-9.75	*<.001*	5.65	4.11-7.76	*<.001*
Infrequent	2.81	2.11-3.74	*<.001*	3.61	2.52-5.18	*<.001*	3.12	2.28-4.26	*<.001*
No	1			1			1		

*P-values *are typed in italic when significant at the 5% level

**Table 5:** Most likely profiles of citizens using the Internet to get health information to help dealing with a consultation

	**Find health information to help decide whether to consult a health professional**		**Find health information prior to an appointment**		**Find information after an appointment with health professionals**	
			
	**General population**	**Health Internet users**	**General population**	**Health Internet users**	**General population**	**Health Internet users**
			
Gender						
			
Age	15-25	15-25	15-25	26-35	26-35	
			
Education	higher education		higher education	higher education	higher education	higher education
				
Kids at home (< 18)						
				
Place of living			main city	main city	main city	main city
				
Work situation						
						
Disable or with diagnosis						
						
Relative disable or with diagnosis						
						
Subjective health						
				
At least one consultation in the last year			yes	yes	yes	yes
			
Interact with a HP never meet face-to-face	infrequent	infrequent	infrequent	infrequent	infrequent	infrequent
			
Participate in forums	established	established	infrequent	infrequent	established	established
			
Order medicines	established	established	established	established	established	established
			
Read about health or illness	established	established	established	established	established	established

**Table 6:** Factors with impact on citizens' behaviours toward health professionals and health system

	Suggestions or queries on diagnosis or treatment to health professional			Changing the use of medicine without consulting a health professional			Making, cancelling or changing na appointment with health professional		
	Oddsratio	CI (95%)	*P*-value	Oddsratio	CI (95%)	*P*-value	Oddsratio	CI (95%)	*P*-value
**Male**	0.88	0.76-1.01	0.072	1.16	0.87-1.55	0.323	1.11	0.91-1.37	0.296
Female	1								
**Age**			*0.034*			0.499			*0.010*
15-25	1.59	1.02-2.47	*0.04*	0.96	0.44-2.08	0.916	1.36	0.75-2.47	0.304
26-35	1.68	1.1-2.57	*0.016*	0.68	0.32-1.46	0.327	1.09	0.61-1.93	0.778
36-45	1.38	0.9-2.13	0.14	0.61	0.28-1.34	0.22	0.7	0.38-1.27	0.24
46-55	1.27	0.83-1.95	0.279	0.66	0.31-1.42	0.289	0.95	0.53-1.71	0.873
56-65	1.18	0.76-1.81	0.46	0.61	0.28-1.35	0.226	0.82	0.45-1.49	0.511
66-80	1			1			1		
**Education**			0.168			0.379			0.370
Higher education	1.21	0.96-1.52	0.109	0.87	0.54-1.38	0.547	0.89	0.64-1.23	0.48
A-Level	1.22	0.99-1.5	0.065	1.1	0.73-1.65	0.649	1.05	0.79-1.4	0.724
Below A-Level	1			1			1		
**Kids at home (< 18)**	1.03	0.88-1.21	0.676	1.22	0.88-1.67	0.231	1.15	0.92-1.43	0.223
No kids at home	1			1			1		
**Place of living**			0.130			0.727			0.150
City	1.09	0.84-1.4	0.53	0.86	0.53-1.41	0.556	1.26	0.87-1.82	0.214
Minor city or suburbs of a big city	1.05	0.81-1.36	0.703	0.76	0.46-1.26	0.284	1.02	0.7-1.49	0.906
Village	1.28	0.98-1.67	0.065	0.88	0.53-1.47	0.633	0.96	0.65-1.41	0.82
Rural/scattered house	1			1			1		
**Work situation**			0.147			0.119			0.310
Student	0.8	0.6-1.08	0.144	0.58	0.33-1.04	0.067	0.77	0.51-1.14	0.186
Working	1.04	0.85-1.29	0.691	0.99	0.66-1.49	0.967	1	0.75-1.33	0.993
Not at work	1								
**Disable or with diagnosis**	1.03	0.82-1.29	0.813	1.12	0.74-1.71	0.588	1.26	0.93-1.7	0.129
No	1			1			1		
**Relative disable or with diagnosis**	1.12	0.95-1.31	0.182	0.97	0.7-1.34	0.85	1.15	0.92-1.43	0.221
No	1								
**Subjective health**			*<.001*			*0.030*			0.466
Good	0.48	0.32-0.74	*<.001*	0.71	0.34-1.48	0.362	0.82	0.48-1.4	0.478
Fair	0.69	0.45-1.06	*0.09*	1.13	0.54-2.35	0.752	0.95	0.55-1.64	0.86
Bad	1			1			1		
**One or more visits to GP in the previous year**	1.75	1.42-2.15	*<.001*	1.26	0.8-1.98	0.311	1.77	1.26-2.49	*<.001*
No visits to GP in the previous year	1			1			1		
**Use of the Internet to interact with unknown HP**			*0.026*			*0.041*			0.132
Established	1.19	0.88-1.62	0.257	0.88	0.5-1.55	0.655	0.8	0.53-1.21	0.292
Infrequent	1.27	1.06-1.52	*0.009*	1.5	1.07-2.11	*0.019*	1.19	0.94-1.52	0.152
No	1			1			1		
**Use of the Internet to participate in forums or self help groups**			*<.001*			*0.002*			*<.001*
Established	1.75	1.31-2.35	*<.001*	2.34	1.48-3.7	*<.001*	1.73	1.21-2.47	*0.003*
Infrequent	1.47	1.21-1.78	*<.001*	1.04	0.7-1.55	0.844	1.52	1.18-1.97	*0.001*
No	1			1			1		
**Use of the Internet to order medicines or other products related to health**			*0.002*			0.151			*<.001*
Established	1.9	1.31-2.74	*<.001*	1.75	1.02-3.03	*0.044*	2.16	1.43-3.25	*<.001*
Infrequent	1.08	0.88-1.31	0.467	1.03	0.69-1.54	0.869	1.65	1.28-2.12	*<.001*
No	1			1			1		
**Use of the Internet to read about health and illness**			*<.001*			0.331			0.052
Established	2.59	1.9-3.52	*<.001*	1.6	0.81-3.18	0.174	1.7	1.04-2.78	*0.033*
Infrequent	1.57	1.16-2.14	*0.004*	1.41	0.72-2.78	0.318	1.45	0.89-2.36	0.14
No	1			1			1		

*P-values *are typed in italic when significant at the 5% level

**Table 7:** Most likely profiles of citizens making suggestions or queries to health professionals or taking vital decisions

	**Suggestions or queries on diagnosis or treatment to health professional**	**Changing the use of medicine without consulting a health professional**	**Making, cancelling or changing an appointment with health professional**
			
Gender			
			
Age	26-35		
			
Education			
			
Kids at home (< 18)			
			
Place of living			
			
Work situation			
			
Disable or with diagnosis			
			
Relative disable or with diagnosis			
			
Subjective health	poor or very poor		
			
At least one consultation in the last year	yes		yes
			
Interact with a HP never meet face-to-face	infrequent	infrequent	
			
Participate in forums	established	established	established
			
Order medicines	established		established
			
Read about health or illness	established		

Analyses were done in the total sample and in the sub-sample of those that have used the Internet for health-related matters. Study of the total sample enabled generalization of the results for the countries' populations, whereas study of the sub-sample led to a better understanding of the behaviour and profiles of Internet users for health or illness purposes.

## Results

In 2007 we see that an estimated 33.9% of the citizens in the seven countries have turned to the Internet to find health information to decide whether to consult a health professional, 25.6% to find health information prior to an appointment and 29.2% to find health information after an appointment (Table 1), corresponding to estimated mean increases of 9.2%, 5.6%, and 7.0%, respectively, from 2005 to 2007. The highest levels are found in Denmark where, in 2007, an estimated 46.1% of the population report having used the Internet to search for health information to help them decide whether to consult a health professional, 34.1% to find health information prior to an appointment with an health professional and 35.7% to find information after an appointment with health professionals. The lowest levels in 2007 are found in Portugal, with an estimated 18.9%, 15.6% and 17.7%, respectively.

Regarding specific behaviours somehow related to the authority and autonomy of health professionals and the use of health system, we see that an estimated 22.0% of the citizens in these seven countries have made suggestions or queries on diagnosis or treatment to a health professional, 3.1% have changed the use of medicine without consulting a health professional and 7.1% have made/cancelled/changed a consultation as a result of health information from the Internet (Table 2), representing estimated mean increases of 5.0%, 0.7% and 1.8%, respectively, from 2005 to 2007. The highest percentages in 2007 are found in Poland (28.1, 3.5 and 11.1) and Latvia (27.6, 6.2 and 9.9) while the lowest percentages are found in Greece (15.2, 0.9 and 3.0) and Portugal (15.1, 2.0 and 2.8).

Results from logistic regressions are reported in Table 3, Table 4 and Table 6. Table 5 summarizes the information in Table 3 and Table 4 and Table 7 summarizes the information in Table 6, reporting the variables with a *P*-value multivariate <.05.

In the seven countries participating in the study, the citizen using the Internet to find health information to support the decision whether to consult a health professional (Table 3 and Table 5) is most likely someone under 25 with higher education, followed by those aged 25-34 years old. She/he has used the Internet to interact with a health professional never met face-to-face on an infrequent base, but is a heavy user of the technology to participate in health-related forums, order medicine and read about health or illness. When we limit the analysis to the sub-group of Internet users for health of illness matters (Table 4 and Table 5), the most significant changes in the profile of the most likely user is that level of education is no longer a discriminating factor. Those using the Internet to get health information prior to a medical appointment among the general population are also most likely under 25, have some higher education, live in a big city, have visited a doctor at least once in the year before the survey and have used the four online health-related services under investigation, although more to order medicines and read health websites than to interact with an unknown doctor on the web or to participate in forums. In the sub-group, most of the characteristics remain, but the most likely user is older, being in the age level 26-35. The citizens turning to the Internet for information about health or illness after a consultation, both among the general population and in the subset of those using the Internet for health matters, are most likely highly educated men and women aged 26-35 years old living in main cities, that have visited a doctor at least once in the year before the survey. They have used the Internet often or very often to participate in health forums, order medicines and read on websites and infrequently to interact with an unknown doctor.

Regarding more specific behaviours toward health professionals and health systems driven by health information from the Internet (Table 4 and Table 5), we can say that the citizen making suggestions or queries on diagnosis or treatment to health professionals is most likely someone aged 26-35 years old feeling in poor or very poor health, that has visited a doctor at least once in the year before the survey and has used the Internet at least once a week to participate in forums, to order medicines and to read about health or illness, and less frequently to interact with a web doctor she/he has never met face-to-face. The profile of the citizen changing the use of a medicine without consulting a doctor is not easy to draw, as we can only state that he/she is more likely to be someone that has used the Internet at least weekly to participate in health forums and less frequently to interact with a web doctor she/he has never met face-to-face. The citizen (re)scheduling a consultation as a result of heath information coming from the Internet is typically someone that has visited a doctor at least once in the year before the survey and who has used the Internet at least once a week to participate in forums and to order medicine.

## Discussion

To the best of our knowledge, this work represents the first attempt to draw the profiles of typical Internet empowered citizens in Europe concerning health. The results expand the conclusions of recent studies [5,7,49-51].

The most important finding of our study is that the Internet does have an impact on the way citizens handle a consultation and on their behaviour towards health professionals and health systems. This is particularly interesting when we realize that, in the 18 months between the two surveys, there were statistically significant increases in the number of citizens in these countries using the Internet to prepare for a consultation and to find health information after an appointment (e.g., second opinion) and using online health information to query the doctor and to take important health decisions (Tables 1 to 2).

Deepening the analysis, we see that such behaviours have different manifestations in different countries and that they are not well predicted neither by the level of use of the Internet for health matter or by its increase from 2005 to 2007 in a specific country. In fact, Danes are the ones that used the Internet the most to help them deal with a consultation in 2007, but increases in the 18 months are lower than in the case of Greeks that were responsible for the highest increases. Yet, Denmark was the country with the highest level of Internet use for health matters in 2007 while Greece ranked last and showed the second lowest increase from 2005 to 2007 [29]. On the other hand, Portuguese are the ones that used the media the least to help them deal with a consultation, but increases while modest where higher than in Norway, the country that ranked second in Internet use for health matters in 2007.

Regarding specific behaviours toward health professionals and health systems driven by online health information, we see that the countries that show the highest growths in the number of Internet health users (Germany, Poland and Latvia) [29] are also those with the highest numbers of assertive patients, making suggestions or queries on diagnosis or treatment to health professionals based on health information they got on the Internet, and those that show the highest growths in this variable between 2005 and 2007. Greece and Portugal, with increases in the number of Internet health users similar to Denmark and higher than Norway [29], turn to be the countries where less citizens query the health professionals due to online health information.

The fact that the citizen using the Internet to find health information to help her/him deal with a consultation is most likely a young, highly educated person is not a surprise, considering that this is also the case of those using the media to find generic health information in many of the researched countries [29,52]. What is new is finding out that, both among the general population and within the subgroup of Internet health users, she/he is a heavy participant in online health forums, shows an established behaviour of buying medicines online and uses the media extremely frequently to consult health websites. In fact, the odds of using the media to help decide whether to consult a health professional and to get a second opinion increase with further use of these three sources of online health information. On the other hand, interacting with a health professional never meet face-to-face is something that these citizens only do on an infrequent base. Interestingly, using the Internet to document themselves before an appointment seems to be a more solitaire endeavour, with only an infrequent interactive component. Researching the reasons and the situations behind such behaviours might be important to inform health professionals and policies makers.

Heavy use of the Internet to participate in health forums, buy medicines and read websites and infrequent interaction with unknown web doctors seem to lead to more assertive patients during the consultation, especially those feeling in fragile health condition. While being more mature than those typically using the media to deal with a consultation, this patient does not necessarily have a high level of formal education what might lead to difficult situations due to poor health literacy and inadequate capacity to dialogue with the doctor.

The profiles of citizens taking critical decisions, such as changing the use of medicine or (re)scheduling an appointment based on health information from the Internet are imprecise, since they cannot be predicted by any of the usual demographic and health variables. The message seems to be that any kind of person might be influenced by health information conveyed in online forums and infrequent interaction with online unknown health professionals and change the use of medicine without consulting a health professional. One may hypothesize that such decisions are being made in very different conditions of previous knowledge and ability to rationalize over whatever information is being made available to them through these two types of interactive web services. On the other hand, online health forums and pharmacies seem to have the potential to lead a heavy user, "all faces" citizen with a history of health system usage to cancel or schedule an appointment with health professional. This might suggest that at least part of these consultations would have the prescription as important or unique subject.

The results of our study clearly show that using the Internet to get information and knowledge about health or illness is currently related to much higher numbers of knowledge-acquirers than decision-makers [53]. This is in line with the results of a Canadian study [54] showing that Internet users seek to develop greater personal mastery of expert knowledge rather questioning the authority of mainstream medicine.

We can hypothesise that such attitude and behaviour may reflect personality traits, but also different personal health experiences, contexts and cultures, as it is suggested by country specific data in our study. It clearly mirrors age or generation group and level of education but not gender and state of health, be it subjective or diagnosed. Similar differences in terms of age, social class and gender have been reported based on a telephone survey [55] conducted in the United Kingdom, aiming to find out how engaged people are in their health care and on 60 interviews to diversified lay people [56] where use of the Internet was not included as a mediating variable.

The division concerning access to health services is well recognized and researched. Underserved and vulnerable populations, who have a high risk of poor health outcomes from serious health problems, frequently have low socioeconomic status, are elderly or have low formal education. They often have limited access to relevant health information, especially information widely available online and low levels of health literacy [57]. Studies show that even in urban settings the use of the Internet for health information is limited among more disadvantaged patient groups [51].

The informed patient will have difficulty in emerging naturally or easily within existing structures and relationships [9]. New strategies are needed to foster the change in paradigm. Actions are required to overcome the digital divide and promote health literacy [58,59]. Reliability and validity of web-based information must be addressed. It is fundamental to assist the public in developing searching [60,61] and appraisal skills but also in balancing self-reliance and compliance with medical management of illness [5] and ensure physicians have adequate communication skills [7] and are prepared for patient questions [62]. New technology such as the Internet may help considerably in this endeavour. Our study shows that online interactive health communities, online pharmacies and unknown web doctors are services deserving special attention, due to their potential to influence important health decisions. Quality criteria need to be developed and effectively communicated.

Doctors in general and family doctors in particular, need to be aware of the importance the Internet has on changing patients' behaviour. Some aspects of this change are clearly evident to doctors on a daily basis, i.e. patients that bring print-outs of information found on the Internet or that refer to such information. However, the Internet may also have a more encompassing and profound role in how patients deal with issues related to health and illness. In some cases, patients can make important decisions without even consulting a doctor. This leaves the doctor out of the loop and effectively changes the doctor's role.

## Study contributions and limitations

At the theoretical level, the main contribution of our work is the setting of a context where the relationship between using the Internet to access health information and attitudes and behaviour towards health professionals and health systems could be analyzed and quantified. Two unidirectional and two bidirectional ways of getting health information through the Internet have been identified. Core activities and crucial moments in the patient-doctor relationship, which the Internet may redefine more or less radically, have been investigated: the need for medical encounter, patient preparation for the consultation and evaluation of its outcome, medication and participation in decision-making about diagnosis or treatment.

Prudence has to be taken when inferring about causation from data that demonstrate an association as it happens with some of the relationships analyzed in this paper. However, regarding the variables "making suggestions or queries on diagnosis or treatment", "changing the use of medicine without consulting a health professional" and "(re)scheduling an appointment with a health professional", it is important to remember that it is the respondents themselves who make the claim about causality between having information on health or illness obtained from the Internet and the aforementioned behaviours.

However, the number of those changing medicine without seeking medical advice and (re)scheduling a medical consultation as a consequence of health information from the Internet is relatively small and results have to be interpreted with caution. Overall, results suggest that the theme deserves more deep and complex investigation.

Results are based on data from seven countries and the study is novel both in its aim and dimension. However, it does not cover all European countries and in some countries the sample of Internet users for health and illness is small, even though it is larger than in many large earlier surveys. In addition, the possibility of generalizing the results may be hindered by the survey response rate and some limitations inherent to telephone surveys. Efforts have been made to validate the eHealth Trends data, comparing it with results of the European Social Survey (ESS) (Sept 2006 - Mar 2007) in similar variable. The European Social Survey (ESS) (Sept 2006 - Mar 2007) used face-to-face interviews at home, with a reported response rate above 60%. We have obtained similar patterns for the two surveys when comparing the frequencies and percentages of the variable subjective health status for five of the seven countries but the ESS did not cover Greece and Latvia [63].

## Conclusion

Information is rarely sufficient for empowerment but few disputes that it is a necessary precondition. Within stated limitations, our study shows that European citizens are using health information they get through the Internet to support their decision on whether they need a medical appointment, prepare for it, and assess its outcome. Having access to health information through online services also gives rise to concrete behaviour toward health professionals and health systems, namely making suggestions or queries on diagnosis or treatment, changing the use of medicine without consulting a health professional and making, cancelling or changing a medical appointment. However, online health information seems to be currently more related to the will or the necessity of being more informed when evaluating the need for a medical appointment and understanding the overall situation and not so much to specific behaviours toward the health professional, such as questioning the doctor during the medical encounter. Moreover our research also shows that some demographic variables are important behaviour predictors and that the studied behaviours increase with more frequent use of the Internet for health related matters. Doctors need to be aware that the Internet is in the process of profoundly changing the doctor-patient relationship, and in particular on what grounds and how patients make decisions about their own health and illness.

## Competing interests

The authors declare that they have no competing interests.

## Authors' contributions

SS and BL have contributed to conception and design, acquisition of data, analysis and interpretation of data, drafting or revising the manuscript critically for important intellectual content, and final approval of the version to be published. CEC and MMB-F have contributed to conception and design, acquisition of data, revising the manuscript critically for important intellectual content, and final approval of the version to be published. H-UP and RW have contributed to conception and design, acquisition of data, revising the manuscript and final approval of the version to be published. All authors have read and approved the final manuscript.

## Pre-publication history

The pre-publication history for this paper can be accessed here:

http://www.biomedcentral.com/1471-2296/12/20/prepub
